# Supplementary figures and images for: ULK2 suppresses ovarian cancer cell migration and invasion by elevating IGFBP3
Source: PeerJ. 2024 Jun 28;12:e17628. doi: 10.7717/peerj.17628 (PMC11216209; doi:10.7717/peerj.17628)

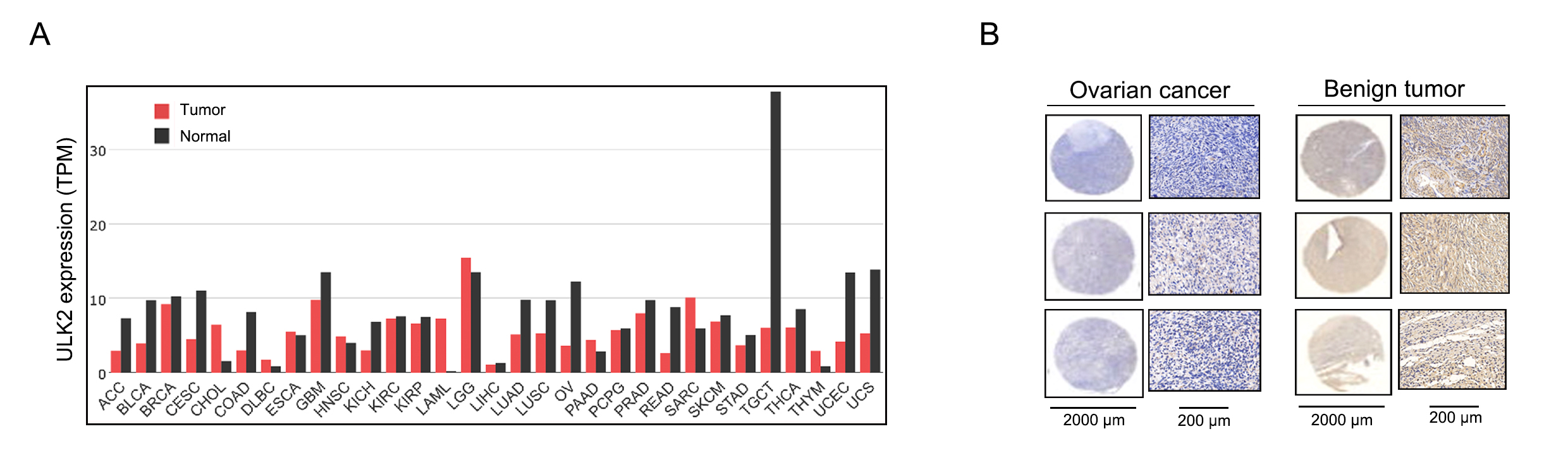

Supplement: Supplemental Information 1 — A. The ULK2 gene expression profile across 31 different types of tumor samples and the normal control tissues. Data were gained from GEPIA database. TPM: Transcripts per million. B. The images depict ULK2 expression detected through immunohistochemical staining in ovarian cancer tissues and benign ovarian tumor samples. [file peerj-12-17628-s001.jpg]

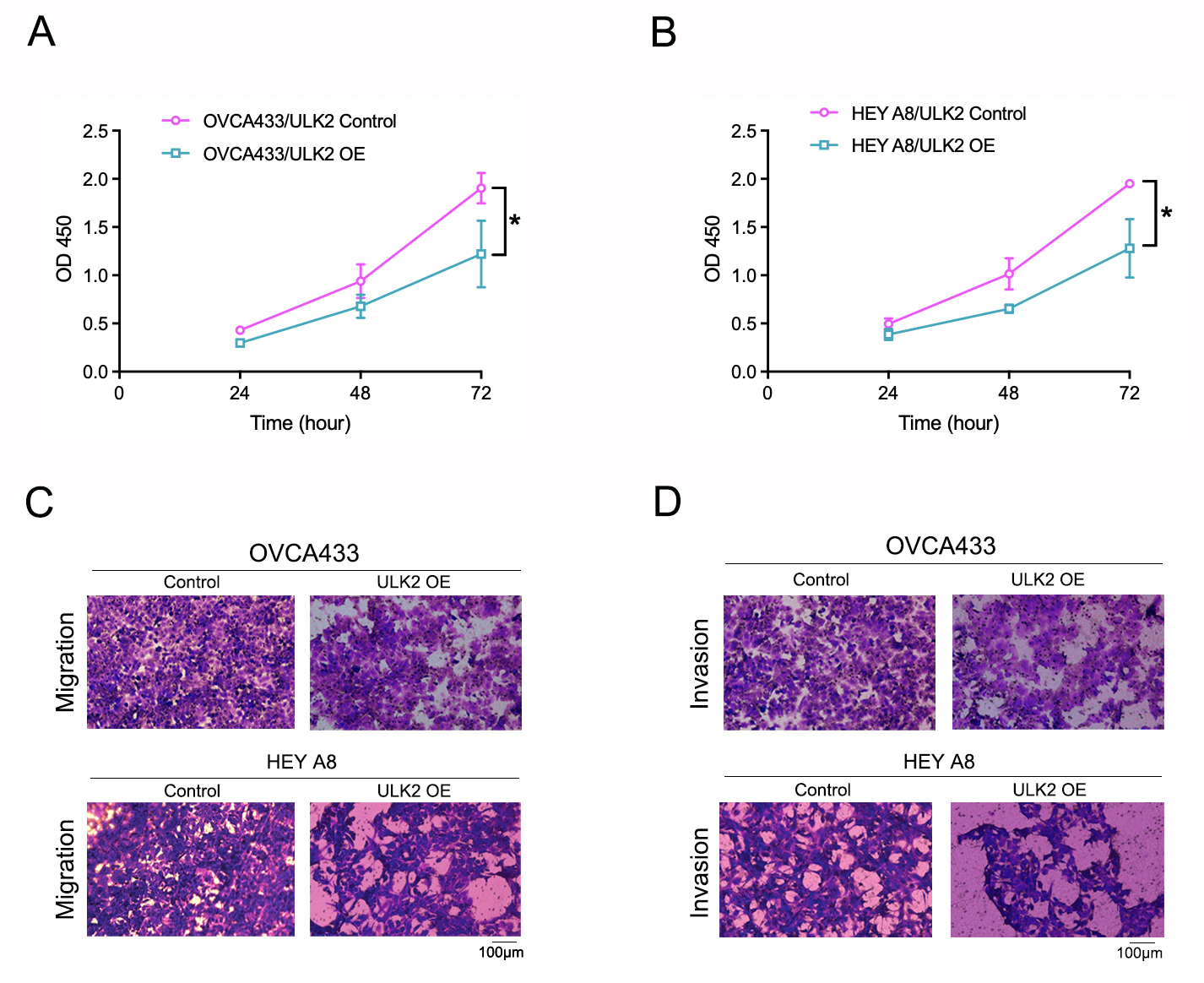

Supplement: Supplemental Information 2 — A. The impact of ULK2 overexpression on the proliferation of OVCA433 ovarian cancer cells was evaluated using the Cell Counting Kit-8 (CCK-8) assay. B. The growth of HEY A8 ovarian cancer cells prompted by upregulation of ULK2 was assessed employing CCK-8 assay. C. Figures illustrating the results of the Transwell migration assay following ULK2 overexpression in ovarian cancer cells were presented. D. The images depicting Transwell invasion assay post- ULK2 overexpression were provided. [file peerj-12-17628-s002.jpg]

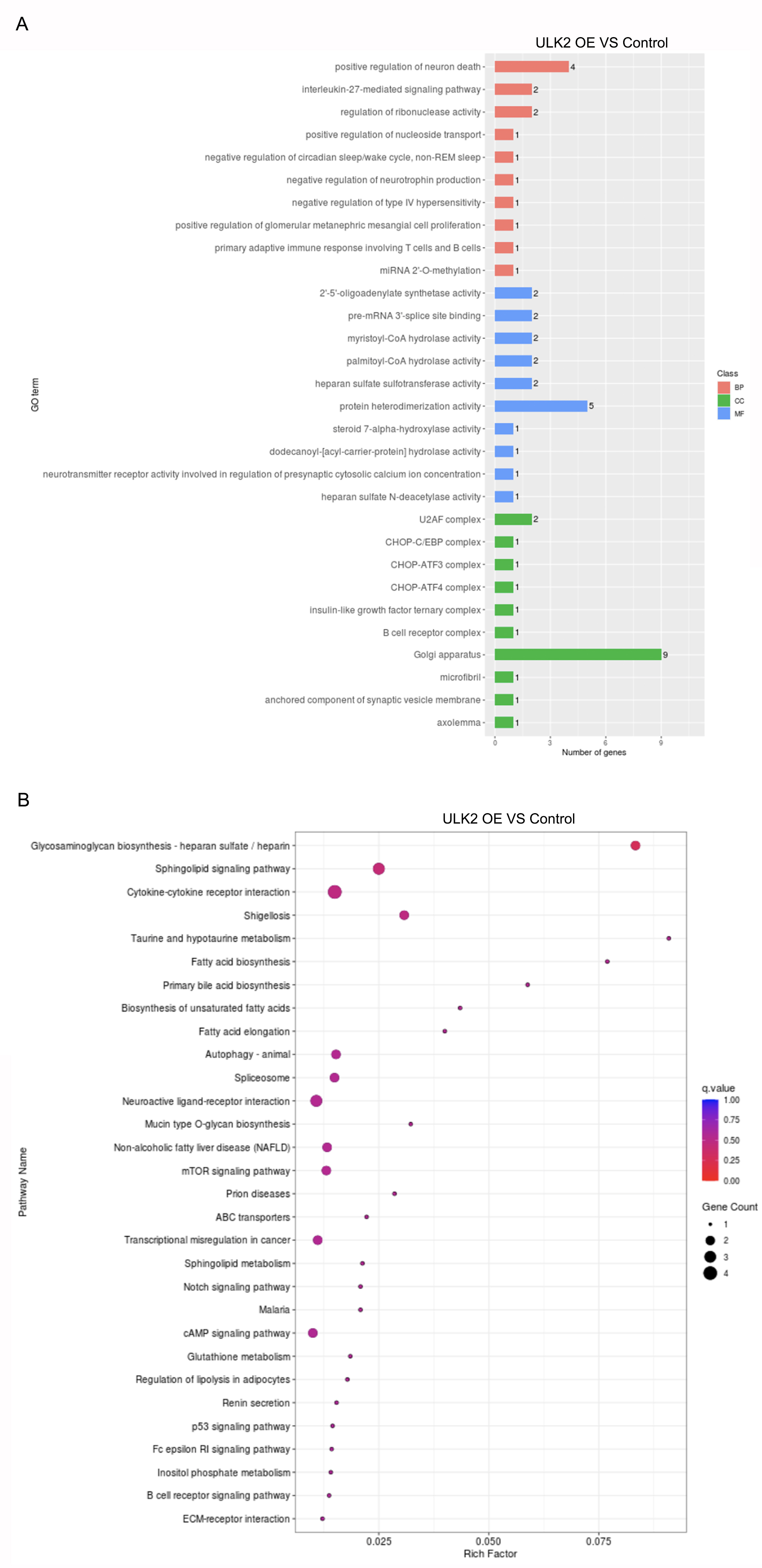

Supplement: Supplemental Information 3 — A. Gene ontology (GO) analysis was conducted on the RNA-sequencing data following ULK2 overexpression. B. RNA-sequencing data was analyzed using KEGG pathway analysis subsequent to the upregulation of ULK2. [file peerj-12-17628-s003.jpg]

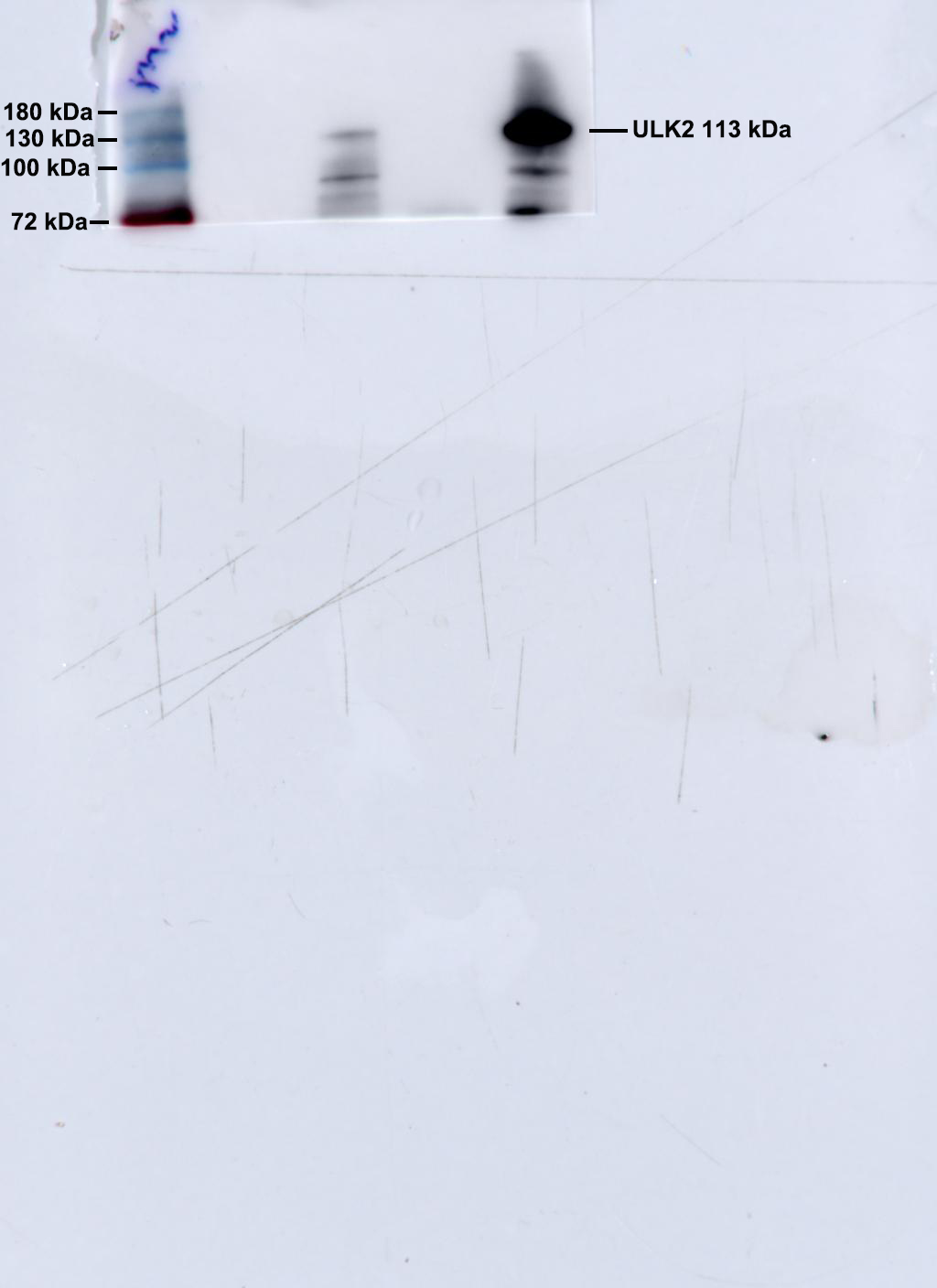

Supplement: Supplemental Information 7 [file peerj-12-17628-s007.zip › Figure 3-lable/ULK2 -repeat.jpg]

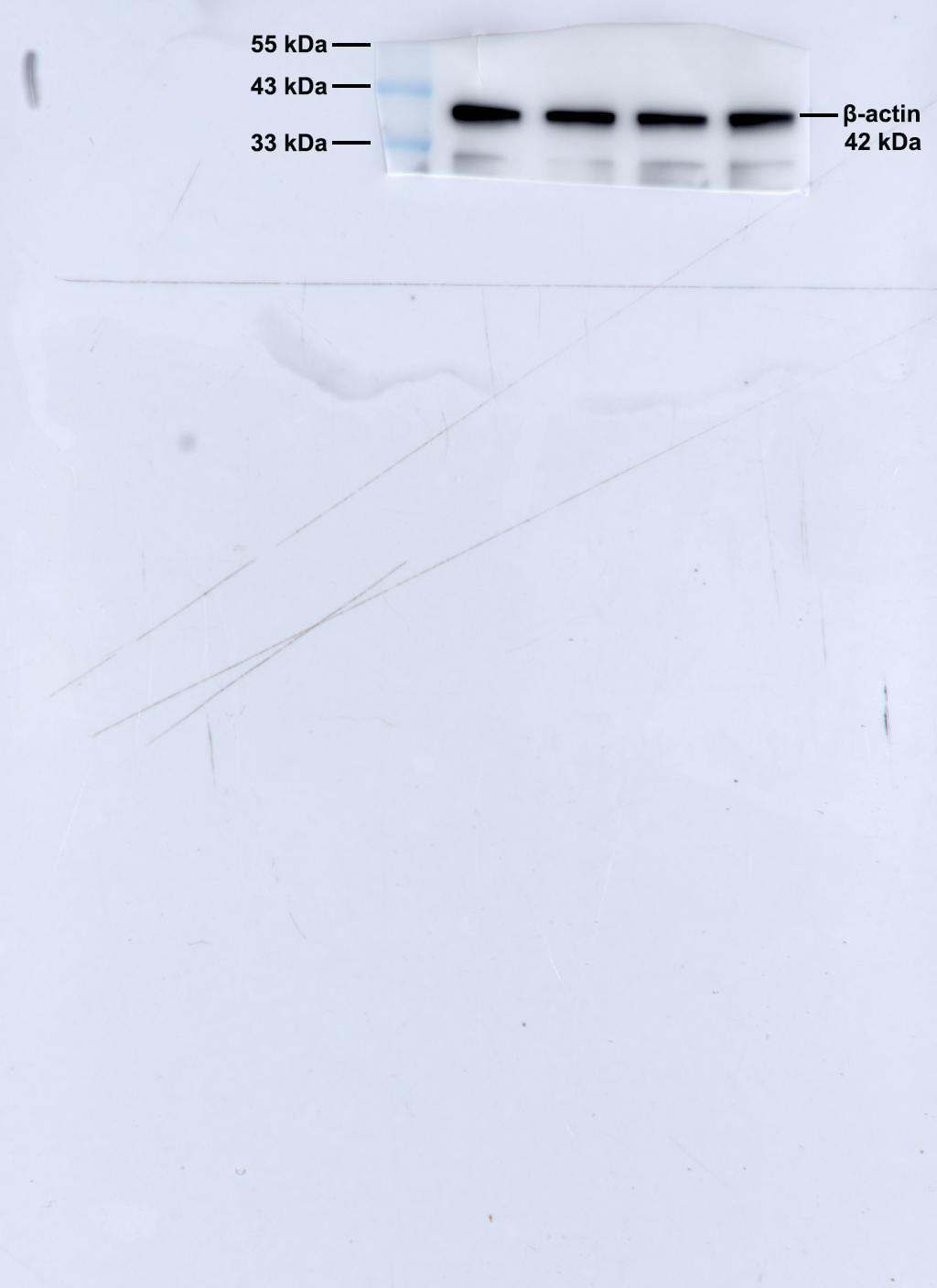

Supplement: Supplemental Information 7 [file peerj-12-17628-s007.zip › Figure 3-lable/actin -repeat.jpg]

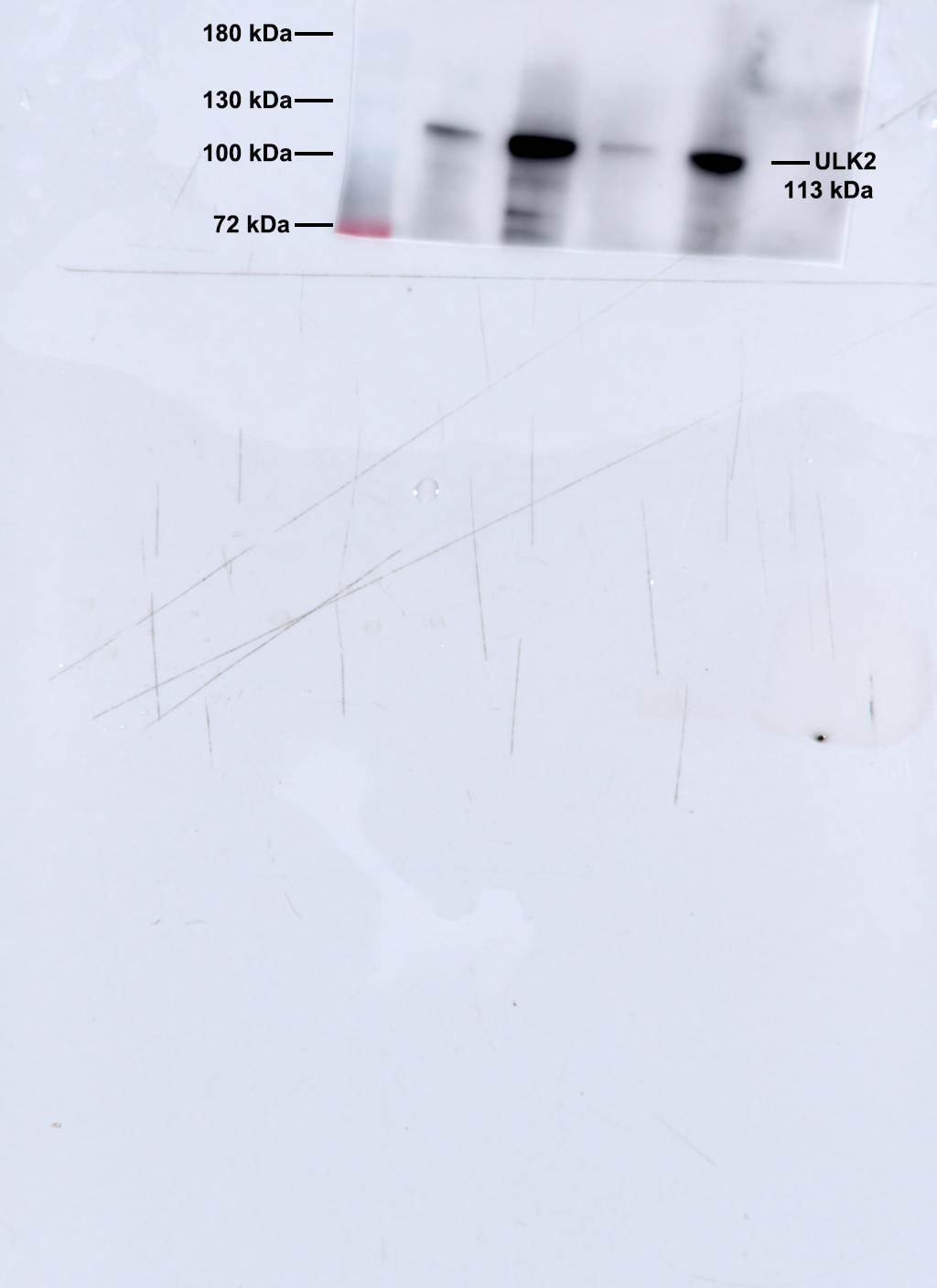

Supplement: Supplemental Information 7 [file peerj-12-17628-s007.zip › Figure 3-lable/ULK2-raw data.jpg]

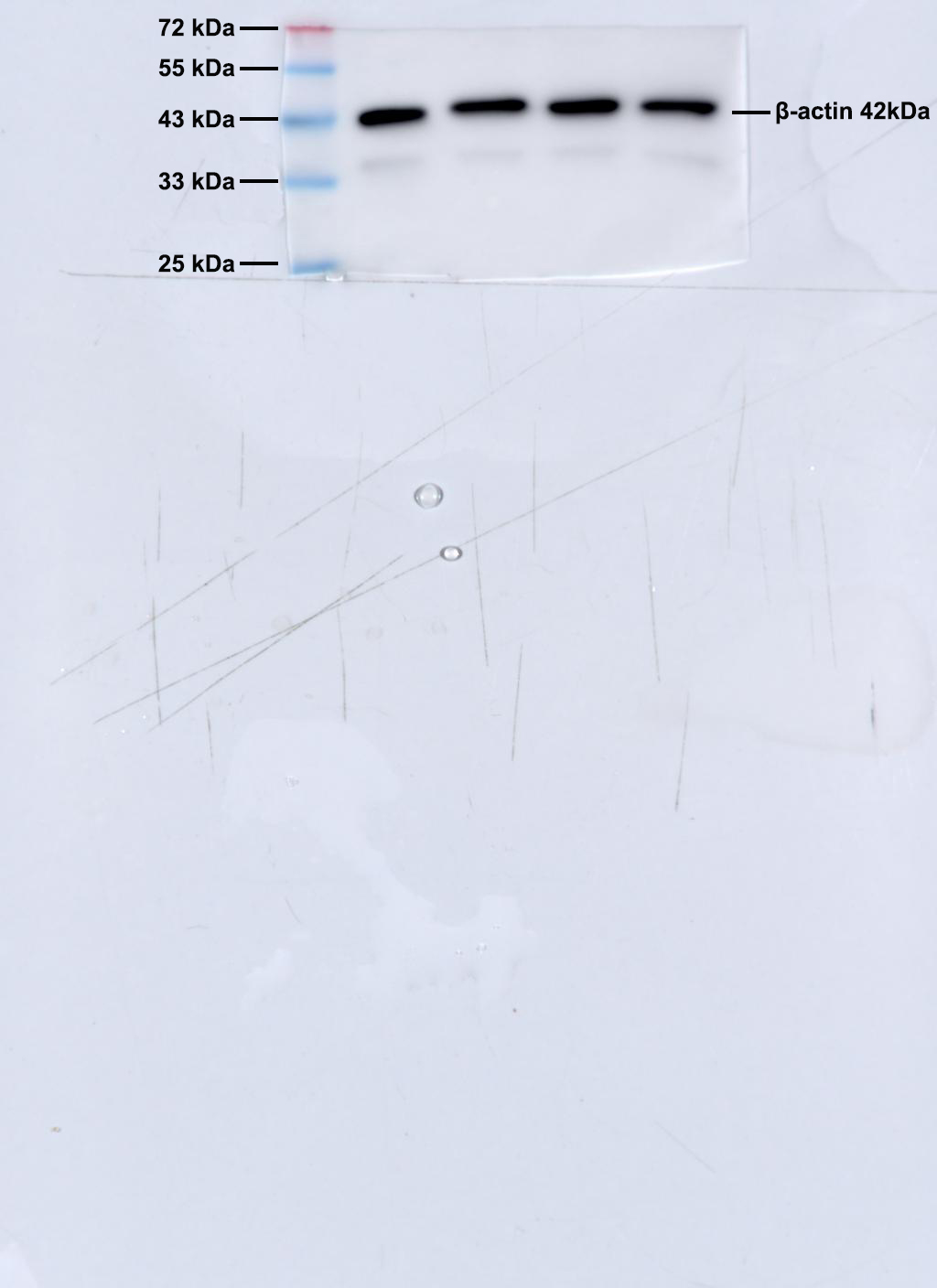

Supplement: Supplemental Information 7 [file peerj-12-17628-s007.zip › Figure 3-lable/actin -raw data.jpg]
