# Supplementary material for: ULK2 suppresses ovarian cancer cell migration and invasion by elevating IGFBP3
Source: PeerJ. 2024 Jun 28;12:e17628. doi: 10.7717/peerj.17628 (PMC11216209; doi:10.7717/peerj.17628)
Supplement: Supplemental Information 4 [file peerj-12-17628-s004.docx]

**Supplementary Table 1. Abbreviations of the various tumor names.**

|  | **abbreviation** | **Full name of the tumor** |
| --- | --- | --- |
| 1 | ACC | Adrenocortical carcinoma |
| 2 | BLCA | Bladder urothelial carcinoma |
| 3 | BRCA | Breast invasive carcinoma |
| 4 | CESC | Cervical squamous cell carcinoma and endocervical adenocarcinoma |
| 5 | CHOL | Cholangio carcinoma |
| 6 | COAD | Colon adenocarcinoma |
| 7 | DLBC | Lymphoid neoplasm diffuse large B-cell lymphoma |
| 8 | ESCA | Esophageal carcinoma |
| 9 | GBM | Glioblastoma multiforme |
| 10 | HNSC | Head and neck squamous cell carcinoma |
| 11 | KICH | Kidney chromophobe |
| 12 | KIRC | Kidney renal clear cell carcinoma |
| 13 | KIRP | Kidney renal papillary cell carcinoma |
| 14 | LAML | Acute myeloid leukemia |
| 15 | LGG | Brain lower grade glioma |
| 16 | LIHC | Liver hepatocellular carcinoma |
| 17 | LUAD | Lung adenocarcinoma |
| 18 | LUSC | Lung squamous cell carcinoma |
| 19 | OV | Ovarian serous cystadenocarcinoma |
| 20 | PAAD | Pancreatic adenocarcinoma |
| 21 | PCPG | Pheochromocytoma and paraganglioma |
| 22 | PRAD | Prostate |
| 23 | READ | Rectum adenocarcinoma |
| 24 | SARC | Sarcoma |
| 25 | SKCM | Skin cutaneous melanoma |
| 26 | STAD | Stomach adenocarcinoma |
| 27 | TGCT | Testicular germ cell tumors |
| 28 | THCA | Thyroid carcinoma |
| 29 | THYM | Thymoma |
| 30 | UCEC | Uterine corpus endometrial carcinoma |
| 31 | UCS | Uterine carcinosarcoma |
