# Supplementary material for: ULK2 suppresses ovarian cancer cell migration and invasion by elevating IGFBP3
Source: PeerJ. 2024 Jun 28;12:e17628. doi: 10.7717/peerj.17628 (PMC11216209; doi:10.7717/peerj.17628)
Supplement: Supplemental Information 5 [file peerj-12-17628-s005.docx]

**Supplementary Table 2. Clinical characteristics of patients with epithelial ovarian cancer and the prognosis analysis using Cox‐regression model.**

| Characteristics | Patients N (%) | H-score | Univariate | Multivariate |  |
| --- | --- | --- | --- | --- | --- |
| **All patients** | | 80 (100) | 19.25±19.05 | P | P |
| Age (years) | |  |  |  |  |
| ≤60 | | 59 (73.75) | 19.24±19.80 |  |  |
| ＞60 | | 21 (26.25) | 19.28±16.77 | 0.454 |  |
| **FIGO Stage** | |  |  |  |  |
| I ~ II | | 40 (50.00) | 26.25±22.48 |  |  |
| III ~ IV | | 40 (50.00) | 12.26±11.08 | **0.000** | **0.008** |
| **Grade** | |  |  |  |  |
| Low ~ Moderate | | 24 (30.00) | 18.98±18.64 |  |  |
| High | | 56 (70.00) | 19.37±19.23 | 0.776 |  |
| **Histology** | |  |  |  |  |
| Serous | | 62 (77.50) | 18.89±18.96 |  |  |
| Other | | 18 (22.50) | 20.50±19.31 | **0.022** | **0.712** |
| **H-score** | |  |  |  |  |
| Low | | 40 (50.00) | 5.38±2.94 |  |  |
| High | | 40 (50.00) | 33.12±18.24 | **0.002** | **0.035** |
|  | |  |  |  |  |
